# Purification, Characterization, and Effect of Thiol Compounds on Activity of the *Erwinia carotovora* L-Asparaginase

**DOI:** 10.4061/2010/165878

**Published:** 2009-11-01

**Authors:** Suchita C. Warangkar, Chandrahas N. Khobragade

**Affiliations:** Biotechnology Research Laboratory, School of Life Sciences, Swami Ramanand Teerth Marathwada University, Nanded 431606, India

## Abstract

L-asparaginase was extracted from *Erwinia carotovora* and purified by ammonium sulfate fractionation (60–70%), Sephadex G-100, CM cellulose, and DEAE sephadex chromatography. The apparent Mr of enzyme under nondenaturing and denaturing conditions was 150 kDa and 37 ± 0.5 kDa, respectively. L-asparaginase activity was studied in presence of thiols, namely, L-cystine (Cys), L-methionine (Met), N-acetyl cysteine (NAC), and reduced glutathione (GSH). Kinetic parameters in presence of thiols (10–400 *μ*M) showed an increase in V_max_ values (2000, 2223, 2380, 2500, and control 1666.7 *μ*moles mg^−1^min^−1^) and a decrease in K_*m*_ values (0.086, 0.076, 0.062, 0.055 and control 0.098 mM) indicating nonessential mode of activation. K_A_ values displayed propensity to bind thiols. A decrease in V_max_/K_*m*_ ratio in concentration plots showed inverse relationship between free thiol groups (NAC and GSH) and bound thiol group (Cys and Met). Enzyme activity was enhanced in presence of thiol protecting reagents like dithiothreitol (DTT), 2-mercaptoethanol (2-ME), and GSH, but inhibited by p-chloromercurybenzoate (PCMB) and iodoacetamide (IA).

## 1. Introduction

L-asparaginase (L-asparagine amino hydrolase, E.C.3.5.1.1) catalyzes the hydrolysis of L-asparagine into L-aspartic acid and ammonia [1–5]. L-asparaginase from *E. coli* and *E. chrysanthemii* has been used as chemotherapeutics in Acute Lymphoblastic Leukemia (ALL) for the past three decades [6]. The L-asparaginases from *E. coli* and *Erwinia* possess different immunological specificities and offer an important alternative therapy if a patient becomes hypersensitive to one of the enzymes [7]. The comparison of the two enzymes leads to the conclusion that *E. coli*-asparaginase can be recommended for first-line therapy, reserving *Erwinia*-asparaginase for allergic patients, because most patients allergic to the former are not immediately allergic to the latter [8]. It has been reported that thiol compounds such as N-acetyl cysteine and glutathione, in a fixed concentration potentiates the activity of asparaginase from *Cylindrocarpon obtusisporum* MB-10 [9] and *Pseudomonas stutzeri* MB-405 [10]. Enzyme activation by thiol group especially from glutathione N-acetyl cysteine is not an uncommon event in enzymology. For instance, catalytic activities of cysteine protease from *Schistosoma mansoni* [11], N^*ω*^-phosphoarginine hydrolase from rat liver [12], reticulocytic protein kinase [13], glyoxylase [14], the family C G-protein coupled receptors, that is, the metotropic glutamate receptors were profoundly accelerated by the addition of GSH [15]. The Chloropicrin activation and reductive dechlorination by Glutathione lead to a decrease in the toxicity index as assayed by *Salmonella* mutagenicity test [16]. The molecular mechanism behind the activation effects of enzymes by glutathione have been the scope of the investigations for researchers. In catalysis by N-terminal hydrolase, Glutathione acts as an additional efficient molecule associated with redox changes [17]. Asparaginase activation is considered to be due to the involvement of free thiol group in the enzyme catalysis [18]. The Glutathione molecules can also binds to the G-protein coupled receptor which acts as a potentiator and agonist. Glutathione binds to family C-receptors in part via bonding interactions between the free *α*-amino and *α*-carboxyl groups of the ligands and a set of highly conserved residues in the binding pocket [19, 20].

In this paper, an attempt has been made to isolate asparaginase producing bacteria from soil, to extract, purify, characterize and examine the effect of four thiol compounds on the kinetic properties of asparaginase obtained from *E. carotovora*. We have investigated the effects of various concentrations of L-cystine (Cys), L-methionine (Met), N-acetyl cysteine (NAC), and reduced Glutathione (GSH) on the maximum velocity (V_max_) of asparagine hydrolysis and the apparent Michaelis-Menten constant (K_*m*_) of the enzyme in order to explain the mechanism of activation of asparaginase activity. The effect of some metal ions is also studied on the activity of L-asparaginase. The activation phenomenon caused due to thiol compounds could be exploited to treat ALL in future.

## 2. Materials and Methods

### 2.1. Screening of Microbial Source

The soil bacteria producing L-asparaginase was primarily screened by using the standard method [21, 22] and then selectively enriched into the growth medium containing yeast extract (1.0%, wt/vol) and L-asparagine (1.5%, wt/vol). A bacterium was grown at 28°C with aeration in Erlenmeyer flasks on a rotary shaker. The cells were harvested by centrifugation near the end of exponential phase of growth and stored at −10°C until needed.

### 2.2. Asparaginase Production

Culture medium was prepared with tryptone (5.0 g/L), yeast extract (5.0 g/L), fructose (1.0 g/L), and K_2_HPO_4_ (5.0 g/L). The initial pH of the medium was adjusted to 7.0. Erlenmeyer flask (250 mL) containing 50 mL of culture medium was inoculated with 1000 *μ*L (2%, v/v) of bacterial suspension with a 0.8 absorbance at 620 nm. For inoculum preparation, two days old slant culture was scraped from agar surface, added to 0.85% sterile saline solution, and mixed until a homogeneous suspension was obtained. The flasks were incubated at 30°C on a rotary shaker at 120 rpm for 2-3 days.

### 2.3. Asparaginase Activity Assay

L-asparaginase activity was assayed by a modified method of Mashburn and Wriston [23]. A 0.1 mL cell suspension or purified enzyme solution, 0.9 mL sodium borate buffer (0.1 M, pH 8.5) and 1 mL L-asparagine (0.04 M) solution were combined and incubated for 10 minutes at 37°C. The reaction was stopped by the addition of 0.5 mL of 15% wt/vol trichloro acetic acid. The reaction contents were centrifuged at 8000 rpm. The supernatant was collected and 0.2 mL supernatant was diluted to 8 mL with distilled water. The resulting mixture was treated with 1.0 mL of Nessler's reagent and 1.0 mL of 2.0 M NaOH. The color reaction was allowed to proceed for 15 minutes before the absorbance at 500 nm was determined. The absorbance was then compared with a standard curve prepared from solutions of ammonium sulfate as the ammonia source. One international unit of L-asparaginase is the amount of enzyme which liberates 1 *μ*mole of ammonia in 1 minute at 37°C [24]. Determinations of protein concentrations were made on whole cell suspension or crude enzyme preparations [25].

### 2.4. Purification of Bacterial L-Asparaginase

Cells were harvested after 24 hours by centrifugation and washed with phosphate buffer (Na_2_HPO_4_, 4.76 g; KH_2_PO_4_, 4.54 g; Triton X-100, 0.125 mL; distilled water to 1 liter; pH adjusted to 7.0). Lysozyme spheroplast formation with subsequent release of L-asparaginase was carried out [26]. Washed cells (300 g) were suspended in 2 liters of 20% sucrose-0.033 M tris hydroxymethyl aminomethane (tris buffer, pH 8.0). Successive treatments of the cells with 0.01 volume of 0.1 M EDTA and lysozyme (5 mg/mL) of suspension were added at 5°C. The suspension was stirred gently, and osmotic fragility was checked at 15-minutes intervals by determining the absorbance at 490 nm. After the decrease in absorbance had ceased, the suspension was centrifuged at 10 000 ×g for 2 hours. To analyze for efficiency of extraction, the spheroplast was resuspended in 2 L of the original sucrose-tris solution. Analyses for enzyme content were made on the resuspended spheroplast and on the supernatant fluid from the final centrifugation [27]. All purification steps were carried out at 4°C unless stated otherwise.

Finely powdered ammonium sulfate was added to the protein to the final concentration of 70% saturation and stirred for 1 hour at 10°C. The mixture was left for 12 hours at 4°C. This was followed by centrifugation at 9000 rpm for 30 minutes at 4°C. The precipitate was dissolved in the phosphate buffer pH 8.5 and dialyzed overnight at 4°C against the same buffer [28].

The 10 mL of diluted supernatant was applied onto the Sephadex G-100 gel filtration column (2.3 cm  × 120 cm), pre-equilibrated with 50 mM tris-HCl buffer pH 8.5 containing 100 mM KCl. The fractions were collected with 1 L of 40 mM sodium phosphate buffer (pH 6.0) at 10 mL/min flow rate (each fraction containing 10 mL). The L-asparaginase activity was assayed by the direct Nesslerization. Fractions containing L-asparaginase activity were pooled and dialyzed against 20 mM sodium phosphate buffer for 24 hours at 4°C.

The dialyzed sample was applied to the column of CM cellulose (2.0 × 12.5 cm) that was pre-equilibrated with 0.01 M sodium phosphate buffer (pH 8.5). It was eluted with the NaCl gradient (0.1–0.5 M) and 0.01 M sodium phosphate buffer (pH 6.0). The fractions were collected and assayed for L-asparaginase activity. Active fractions were collected and used in the subsequent steps of purification.

A chromatography column (2.5 × 25 cm) was packed with DEAE sephadex and equilibrated with 0.l M tris HCl buffer containing 2 mM MgCl_2_ (pH 8.6). The desalted enzyme thus obtained from dialysis was loaded on to the top of the column and eluted with 1 L of linear gradient tris HCl buffer composed of sodium chloride (0.l M). The flow rate of the column was adjusted to 10 mL/min constantly to determine the protein concentration in the eluant (280 nm uv/vis Schimadzu). The 10 mL fractions were pooled and assayed for the protein and enzyme activity.

### 2.5. Electrophoresis

The mass of the native protein was determined by native gel electrophoresis on 10% acryl amide gels in tris-glycine (pH 8.5) using Genei Mini-V 8 × 10 vertical gel electrophoresis system (Banglore Genei, India). Gels were stained with coomassie brilliant blue. Myosin rabbit muscle (205 kDa), phosphorylase b (97.4 kDa), BSA (66 kDa), ovalbumin (43 kDa), carbonic anhydrase (29 kDa), soybean trypsin inhibitor (14.3 kDa), and aprotinin (6.5 kDa) were used as protein standards in native gel, while Phosphorylase b (97.3 kDa), bovine serum albumin (66 kDa), ovalbumin (43 kDa), soyabean trypsin inhibitor (20 kDa), and lysozyme (14.3 kDa) were used as molecular weight markers for Sodium dodecyl sulfate polyacrylamide gel electrophoresis (SDS-PAGE). SDS-PAGE was carried out in 3 mm slab gel of 6% acrylamide in a tris borate buffer pH 7.2 containing 0.1% SDS. The gels were stained with 0.025% coomassie brilliant blue R-250 and destained by 1.0% acetic acid solution [29].

### 2.6. Molecular Weight Determination by Gel Filtration

The molecular weight of the enzyme subunit was estimated by gel filtration chromatography through a column (2.3 cm  × 120 cm) of sephadex G-100 [30]. The column was equilibrated with 50 mM Tris HCl buffer, pH 8.5, containing 100 mM KCl. The column was calibrated with blue dextran and standard molecular weight marker proteins: Bovine Serum Albumin (66 KDa), Egg albumin (45 KDa), Carbonic anhydrase (29 KDa), and Cytochrome c (12.4 KDa). The elution volume (V_e_) of each marker protein and void volume (V_o_) of the column were estimated. A plot of V_e_/V_o_ against log molecular weight was used to determine the molecular weight of L-asparaginase.

### 2.7. Effect of Reagents on L-Asparaginase Activity

The effect of different compounds was tested by preincubation (37°C for 20 minutes) of properly diluted enzyme with the respective reagents. The working concentrations of metal ions and thiol compounds were set at 0.5–10 mM and other thiol compounds were set at 10–400 *μ*M, respectively. After pre incubation, enzymatic assay was performed under optimal conditions, and enzyme activity was expressed as the percentage of the activity observed without additions of metal ions, thiol group blocking reagents, and thiols.

### 2.8. Kinetic Measurements

In all cases, enzymatic activity was assayed under temperature and pH optima. Alternatively data were fitted to the Michaelis-Menten equation to obtain the kinetic constants by applying linear regression. Activation of asparagine hydrolysis was studied in terms of change in values of kinetic parameters (K_*m*_ and V_max_) in presence and absence of thiol compounds. The values of parameters were determined from Lineweaver-Burk plots. The binding constants (K_*A*_) were obtained by plotting K_*m*_/V_max_ versus thiol concentration. Kinetic analyses were also performed in the presence of fixed NAC and GSH concentrations (10–400 *μ*M) using different asparagine concentrations in the range of 0.02 to 0.2 mM. Enzymatic activity was determined as mentioned above taking into account that aspartate formation was linear for 40 minutes, in the presence of GSH and NAC under all conditions studied. On the other hand, Lineweaver-Burk analyses were performed to determine the binding constant (K_*A*_) for both GSH and NAC with the asparaginase interactions. These constants were calculated by using the secondary replots of 1/Δ slope versus 1/[thiol compound] according to Segel [31]. The Δ slope values were obtained from individual Lineweaver-Burk plots. The constants *α* and *β* refer to the fold change in the K_*m*_ and V_max_, respectively, obtained in the presence of nonessential activator. In 1/Δ slope versus 1/[thiol] replots, the *x* and *y* intercepts correspond to −*β*/*α*  K_*A*_ and *β*V_max_/K_*m*_ (*β*-*α*), respectively. By assigning the values for *α* and *β*, K_*A*_ values are estimated accurately.

## 3. Results and Discussions

### 3.1. Screening of Microbial Source

The organism has been characterized as the genus *Erwinia*. It is a gram negative motile rod with peritrichous flagella (0.5 × 1.0–3.0 *μ*m), sometimes occurs singly or in pairs and also forms short chains. On minimal agar slants colonies are shiny, smooth and circular off-white colored. Blood agar was not hemolysed. Growth as well as liquefaction of gelatin was seen in gelatin stabs. The bacillus was negative for cytochrome oxidase activity but positive for catalase activity. It converts nitrate to nitrite. Acid was produced with slight amount of gas from sugars like fructose, galactose, dextrose, and sucrose, and *β*-methyl glucoside. Various organic compounds are utilized as a source of carbon and energy viz citrate, lactate, formate, galacturonate, but tartarate, malate, benzoate, oxalate, and propionate do not serve as source of carbon and energy. There was no pigmentation on minimal agar. Casein and cotton seed oil was hydrolyzed at 37°C in medium which confirms the differentiation in between subspecies of *E. carotovora*.

### 3.2. Purification of Bacterial L-Asparaginase

**Figure 1:**
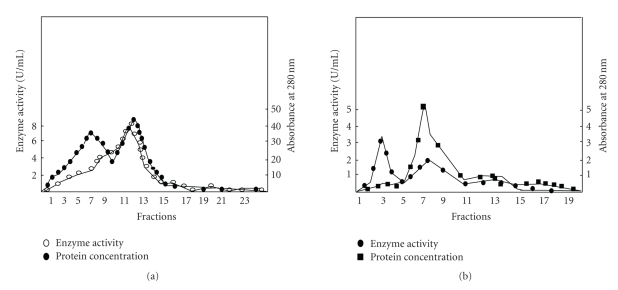
Purification of L-asparaginase from the *E. carotovora*. (a) CM cellulose chromatography of the active fractions collected from the Sephadex G-100 gel filtration column. (b) DEAE cellulose Anion Exchange Chromatography of the active fractions collected from the CM cellulose chromatography column.

L-asparaginase was purified from *E. carotovora*, using spheroplast formation, ammonium sulfate fractionation, sephadex G-100 gel filtration, CM cellulose, and DEAE Sephadex column chromatography. The recovery of enzyme is shown in Table 1. The specific activity of the enzyme increased with every step of purification with a minimum loss in quantity, giving a final recovery of 36.5% (Table 1). After every purification procedure, the peak fractions with the enzyme activity were analyzed using SDS-PAGE. The enzyme precipitated out between 60 and 70% of ammonium sulfate saturation. Removal of salt from the enzyme by Sephadex G-100 was found to be suitable after that step enzyme loss was negligible. Peak fractions of Sephadex G-100 chromatography were pooled together and loaded onto equilibrated CM cellulose column for purification and in the eluant two protein peaks and one enzyme activity peak are obtained (Figure 1(a)). Enzyme activity peak was matched with the second protein peak. Further peak fractions were collected from CM cellulose chromatography and loaded onto equilibrated DEAE Sephadex column for final purification. The elution profile has shown two peaks for proteins and one for enzyme activity. Enzyme activity was detected only in trace amount in the peak I fractions. While peak II has showed lower protein concentration but higher enzyme activity (Figure 1(b)).

### 3.3. Electrophoresis and Molecular Weight Determination

**Figure 2:**
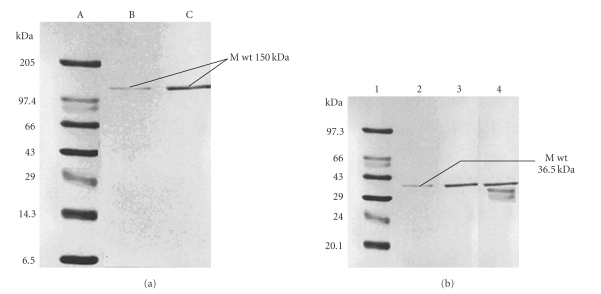
(a) Native PAGE of the purified L-asparaginase from the *E. carotovora*, (i) molecular weight marker proteins, (ii) purified asparaginase 10 *μ*gm/mL, (iii) purified asparaginase 25 *μ*gm/mL. (b) SDS-PAGE of the purified L-asparaginase from the *E. carotovora*, lane 1 molecular weight marker proteins, lane 2 purified asparaginase after DEAE sephadex chromatography, lane 3 purified asparaginase after CM Cellulose chromatography, lane 4 crude preparation.

**Figure 3:**
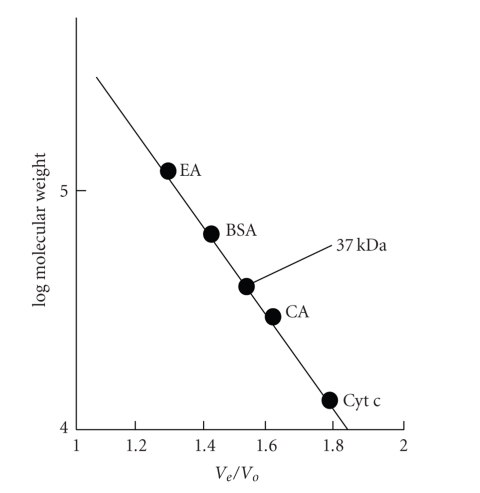
Calibration curve for the determination of molecular weight of L-asparaginase by gel filtration chromatography.

The L-asparaginase from *E. carotovora* is active as homotetramer and its crystal structure (PDB ID 1ZCF) and function have been thoroughly characterized with the approximate molecular mass of 150 kDa as determined by native PAGE (Figure 2(a)) [32, 33]. The molecular mass of enzyme subunit is 36.5 ± 0.5 kDa as observed from SDS-PAGE separation and gel filtration. The SDS-PAGE of the enzyme preparation from different purification steps showed that the resolved electrophoretic bands were progressively improved from the crude extract to the final step of purification. It revealed only one distinctive band that was indicated by the pure preparation of L-asparaginase (Figure 2(b)). Native PAGE has exhibited an approximate molecular weight 150 kDA, which indicates that L-asparaginase purified from *E. carotovora* was homogeneous. SDS-PAGE and gel filtration chromatography indicated that enzyme subunit is one band with approximate molecular weight of 36.5 kDa and 37 kDa, respectively (Figure 3). This value was equal to tumor inhibitory L-asparaginase [32].

### 3.4. Effect of Reagents on L-Asparaginase Activity

L-asparaginase activity was assayed in the presence of different reagents (Table 2). Among the salts tested, considerable loss of activity was observed only with Hg^2+^, Ni^2+^, Cd^2+^, Cu^2+^, Fe^3+^, Mg^2+^ and Zn^2+^, whereas Na^+^ and K^+^ acting somewhat as an enhancer. This was also true for EDTA. The data indicates that asparaginase may not be the metallo-enzyme. Among the amino acids tested, only L-cysteine and histidine stimulate the relative activity, while others had not observable effect. Inhibition of enzyme activity in presence of Hg^2+^, Cd^2+^, and Zn^2**+ **^ might be indicative of essential vicinal sulfhydryl groups of the enzyme for productive catalysis. Furthermore, stimulation of the activation with reducing agents like 2-mercaptoethanol (2-ME), dithiothreitol (DTT), and reduced glutathione (GSH) and inhibition in the presence of thiol group blocking reagents, namely, PCMB (P-chloro mercury benzoate) and IA (iodiacetate) provided additional proof for the role of sulfhydryl groups in the catalytic activity of the enzyme. The enzyme completely lost its activity at 4.0 M urea and only 22% of activity was retained at 3.0 M sodium dodecyl sulfate (SDS).

### 3.5. Kinetic Measurements

The enzyme showed typical Michaelis-Menten kinetics at lower substrate concentrations and the apparent K_*m*_ value for L-asparagine is 0.098 mM. The apparent K_*m*_ and V_max_ values were tentatively determined from both linear and nonlinear regressions using asparagine and glutamine as substrates. The lower apparent K_*m*_ value for L-asparagine indicates that the purified L-asparaginase has higher affinity for the substrate asparagine. The efficiency of substrate utilization was estimated by V_max_/K_*m*_ ratios (Table 3) and the hydrolysis efficiency of asparagine was at least 11 925 fold higher than that of L-glutamine.

### 3.6. Effect of Thiol Compounds on L-Asparaginase

**Figure 4:**
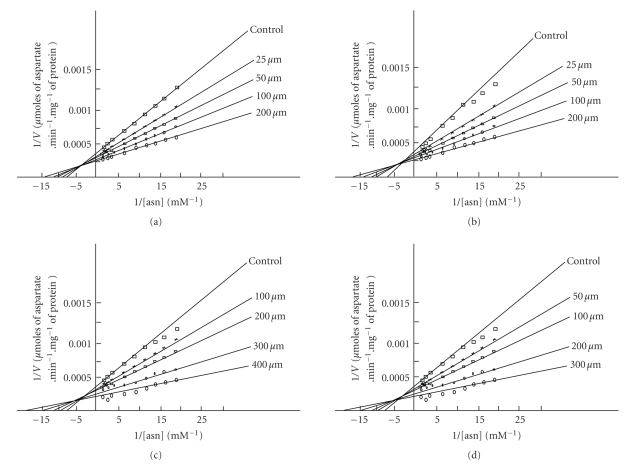
Lineweaver-Burk analyses of activity shown by asparaginase from *E. carotovora* in the presence of different concentrations of Cys (a), Met (b), NAC (c), and GSH (d).

**Figure 5:**
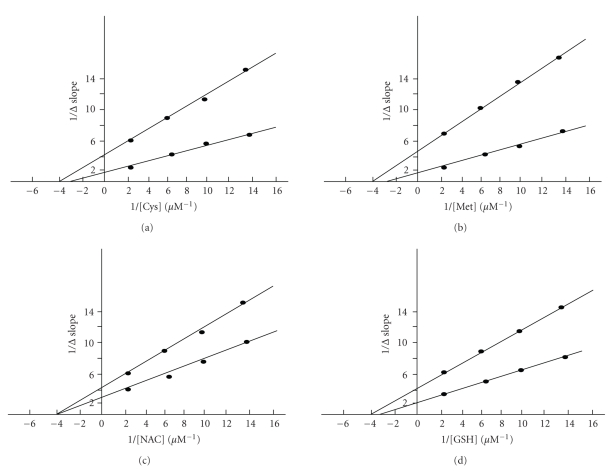
The secondary replots of 1/Δ slope versus 1/[Cys] (a), 1/Δ slope versus 1/[Met] (b), 1/Δ slope versus 1/[NAC] (c), and 1/Δ slope versus 1/[GSH] (d).

**Figure 6:**
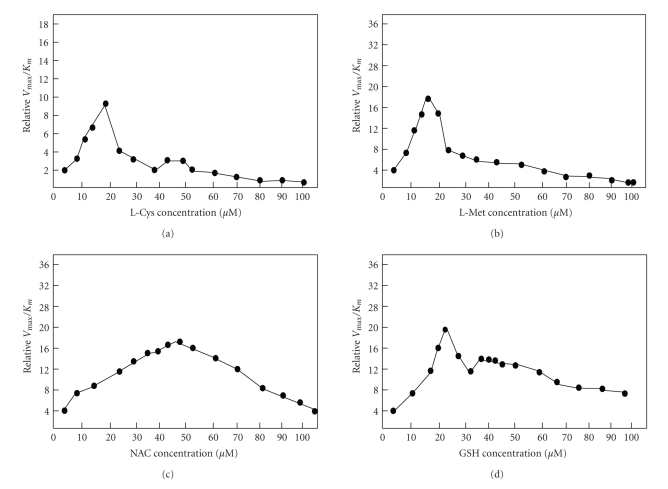
Effect of thiol containing compounds on the relative V_max_/K_*m*_ ratio of L-asparaginase from *E. carotovora*.

The enzyme activity was determined in the presence of different thiol compounds. A lower concentration of GSH and NAC in the medium has exhibited stimulatory effects on L-asparaginase catalytic activity. The activation was fully characterized by performing Lineweaver-Burk analyses using different concentration of Cys, Met, NAC, and GSH (10–400 *μ*M) (Figure 4). An increase in amount of GSH and NAC in the reaction medium revealed an increase in V_max_ and a decrease in K_*m*_ values, which corresponds to a nonessential activation [34, 35]. The secondary replot of 1/Δ slope versus 1/[thiols] (Figure 5) enabled us to know *α*, *β*, and binding constant K_*A*_ values for each thiol compounds (Table 4). The constants *α* and *β* refer to the fold change in K_*m*_ and V_max_, respectively, in the presence of each thiol compound. From Table 4, it is evident that the K_*A*_ value for the enzyme decreases from Cys, Met, NAC, and GSH. As a consequence, the concentration of each thiol compound at which maximum stimulation was achieved was lowest for Cys and Met. This data suggests that relatively specific interaction may take place between the free thiol group and enzyme, as a critical determinant of this interaction as well as activation. Thiol compound binding may lead to decrease in the K_*m*_ which is more evident with GSH (*α* = 0.062). In this sense, *α* value gradually decreases when the thiol group of compound is not free for interaction with enzyme. However, the magnitude of nonessential activation is almost same for every free thiol group containing compounds, taking into account the *β* values (Table 4). The catalytic activity of asparaginase from *E. carotovora* was not further increased, when the concentrations of each thiol compound were increased in the assay medium more than the concentration (400 *μ*M) reported for the nonessential activation. All the data described previously were expressed in terms of relative V_max_/K_*m*_ ratio and plotted versus thiol compound concentrations (Figure 6). For each of the four thiol compounds at the lower concentrations, these replots exhibited positive slope, indicating that GSH and NAC act as activators at these concentrations, while Cys and Met at lower concentrations result in a negative slope indicating inhibition. It is obvious to observe an inverse relationship between the free and bound thiol group of the compounds and the breakpoint in the V_max_/K_*m*_ ratio versus thiol compound concentration plots.

**Scheme 1:**
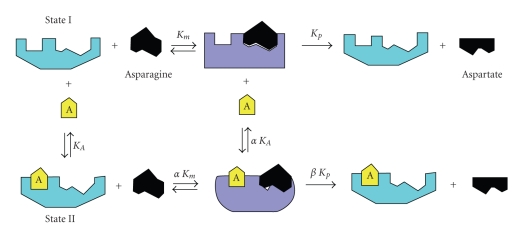
Model for the nonessential activation of asparaginase by thiol containing compounds.

**Scheme 2:**
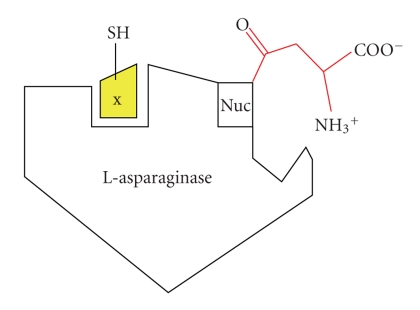
Acyl enzyme intermediate attack by nucleophile Tyr/Thr. The probable acyl enzyme intermediate of asparaginase from *E. coli* where Thr is a nucleophile in the N-terminal position.

Bacterial asparaginases have been the subject of considerable medical interest and are being employed in the therapy of acute lymphoblastic leukemia. Its therapeutic potential is now well established, as it has remarkably induced remission in most of the patients suffering with ALL. A comparative examination of preparations of *Erwinia* L-asparaginase made in USSR and Germany was recommended for clinical use. The catalytic geometry and the secondary structure around the active sites of asparaginase from *E. coli*, *E. carotovora,* and *E. chrysanthemii* are closely similar but their substrate specificity pockets are quite different. To explain the mechanism of asparaginase activation by GSH and NAC, it is to conceive that asparaginase possesses the thiol group binding domain with high affinity towards free-SH group containing effectors. Using this model, the most hydrophilic compounds such as GSH and NAC would bind more effectively to the activator site and convert the asparaginase from one conformation to another conformation, that is, State I to State II (Scheme 1). The later form is catalytically more active than the free form of the enzyme, as is evident from the increase in V_max_/K_*m*_ ratio. The asparaginase activation induced by GSH and NAC can be considered as a case of nonessential activation. In the extensive kinetic analyses of 1/Δ slope versus 1/[thiol compound], values of *β*  V_max_/K_*m*_ (*β*-*α*) are determined, which are approximately same for NAC and GSH, but different for Cys and Met. This supports the hypothesis that all thiol group containing amino acids and compounds may interact with the same activator site on asparaginase. This kind of stimulatory action has been also studied with the enzyme glyoxalase, but it is concentration dependant. The mechanism of NAC and GSH mediated activation in mitogen activated protein kinase was redox dependant and requires reactive oxygen species (ROS) sensitive mechanism for regulation of TNF-*α* biosynthesis [13]. Molecular docking experiments with GSH in G-protein coupled receptor have shown that the peptide binds in a “bent” conformation by protruding the terminal glycine residue upward in the pocket. At the same time, it was also proposed that like free amino acids, GSH acts in conjunction with divalent cation ligands promoted closure of the Venus fly trap domain and initiate receptor activation [15]. This effect was probably the cause of a more flexible enzyme conformation, which has a higher catalytic activity. The authors ascribed this effect to an enzyme conformational change provoked by free thiol group containing amino acids with the enzyme at a locus other than that for substrate binding (Scheme 2). In asparaginase, the lower concentrations of free thiol group containing non polar amino acids should change the enzyme structure to a more active conformation due to a more effective interaction with the hydrophilic activator site. Higher concentrations of NAC and GSH were resulted in an uncompetitive mode of inhibition of enzyme in its activated state. It was proposed that once the activator site of asparaginase is filled and all enzyme molecules are activated, the excess of molecules may compete with enzyme substrate complex (ES) during the deamination step of the hydrolysis, accounting for the uncompetitive inhibition and lowering the turnover.

The whole data of the present work indicate that NAC and GSH may binds to the activator site, which is very hydrophobic pocket nearer to the hydrophilic active site. In the present paper, we have described the extraction, purification, characterization, and activation of asparaginase from *E. carotovora* by GSH and NAC and kinetic events accompanying with the increase of thiol concentration in a reaction medium. This biocatalyst in the presence of thiol compounds will be more useful to improve a potential biotechnological purpose and to increase the catalytic efficiency of asparaginase as the therapeutic agent in the treatment of ALL and other blood system tumors. The comparison of crystal structures, active sites, combined crystallographic, thermal stability, and cytotoxic experiments has shown that *E. carotovora* asparaginase is 30 times less toxic against the human leukemia cell lines. But, denaturing experiments showed that* E. carotovora* asparaginase has decreased thermodynamic stability as compared to the *E. coli* asparaginase and get rapidly inactivated in the presence of urea [33]. Our studies may find some alternative to stablise the structure and function of *E. carotovora* asparaginase by adding thiols.

## Figures and Tables

**Table 1:** L-asparaginase purification summary.

Purification steps	Total activity (IU)	Total Protein (mg)	Specific activity (IU/mg)	Purification fold	Recovery (%)
Spheroplast suspension	6800	5000	1.36	1.0	100
Ammonium sulfate precipitation	5000	1400	3.57	2.62	73
Sephadex G-100 gel filtration	4100	248.9	16.4	12.11	60
CM Cellulose	3172	10.2	310.9	228.6	46
DEAE sephadex	2482	2.4	1034	752.9	36.5

**Table 2:** Influence of different metal ions and reagents on L-asparaginase activity.

Addition	Concentration (mM)	Relative activity (%)
No addition	none	100
Na^+^ (NaCl)	1.0	117
K^+^ (KCl)	1.0	133
Mg^2+^ (MgCl_2_)	1.0	24
Ca^2+^ (CaCl_2_)	1.0	78
Mn^2+^ (MnCl_2_)	1.0	71
Zn^2+^ (ZnCl_2_)	0.5	06
Fe^3+^ (FeCl_3_)	0.5	47
Hg^2+^ (HgCl_2_)	1.0	0
Ni^2+^ (NiCl_2_)	1.0	51
Cu^2+^ (CuCl_2_)	1.0	0
Cd^2+^ (CdCl_2_)	1.0	0
EDTA	0.5	117
L-Cysteine	25	148
L-Histidine	25	114
Glutathione (reduced)	0.5	136
Thiourea	0.5	114
Thioacetic acid	0.5	106
Thioacetamide	0.5	110
2-mercaptoethanol	0.5	112
Dithiothreitol	0.5	86
p-chloro mercury benzoic acid	0.5	0
Iodoacetamide	0.5	0
Sodium dodecyl sulfate	2.0	119
	3.0	22
Urea	2.0	108
H_2_O_2_	1.0	24

**Table 3:** Kinetic constants of partially purified asparaginase.

Substrate	Linear regression (Lineweaver-Burkplot)			Nonlinear regression (Michaelis-Menten equation)		
	K_*m*_	V_max_	V_max_/K_*m*_	K_*m*_	V_max_	V_max_/K_*m*_
	(mM)	(*μ*moles mg^−1^min^−1^)		(mM)	(*μ*moles mg^−1^min^−1^)	
L-asparagine	0.096	1632.6	17,006	0.098	1666.7	17,007
L-glutamine	2.86	4.08	1.426	3.04	4.74	1.559

**Table 4:** Summary of activation parameters and affinity constants of thiol containing compounds to asparaginase.

Thiol compounds	*α*	*β*	K_*A*_	K_*m*_	V_max_	V_max_/K_*m*_
			(mM)	(mM)	(*μ*moles mg^−1^min^−1^)	
control	—	—	—	0.098	1666.7	17,007
Cys	0.589	1.87	293	0.086	2000	23,255
Met	0.138	1.96	229	0.076	2223	29,250
NAC	0.081	2.27	128	0.062	2380	38,387
GSH	0.062	1.89	14.6	0.055	2500	45,454
